# Fabrication of High-Density Out-of-Plane Microneedle Arrays with Various Heights and Diverse Cross-Sectional Shapes

**DOI:** 10.1007/s40820-021-00778-1

**Published:** 2021-12-09

**Authors:** Hyeonhee Roh, Young Jun Yoon, Jin Soo Park, Dong-Hyun Kang, Seung Min Kwak, Byung Chul Lee, Maesoon Im

**Affiliations:** 1 Brain Science Institute, Korea Institute of Science and Technology (KIST), Seoul, 02792 South Korea; 2 Division of Electrical Engineering, College of Engineering, Korea University, Seoul, 02841 South Korea; 3 Micro/Nano Fabrication Center, KIST, Seoul, 02792 South Korea; 4 Division of Bio-Medical Science & Technology, KIST School, University of Science & Technology (UST), Seoul, 02792 South Korea

**Keywords:** Microneedle, Various heights, Cross-sectional shapes, Isotropic etch, Deep reactive ion etching

## Abstract

Out-of-plane microneedle structures are widely used in various applications such as transcutaneous drug delivery and neural signal recording for brain machine interface. This work presents a novel but simple method to fabricate high-density silicon (Si) microneedle arrays with various heights and diverse cross-sectional shapes depending on photomask pattern designs. The proposed fabrication method is composed of a single photolithography and two subsequent deep reactive ion etching (DRIE) steps. First, a photoresist layer was patterned on a Si substrate to define areas to be etched, which will eventually determine the final location and shape of each individual microneedle. Then, the 1st DRIE step created deep trenches with a highly anisotropic etching of the Si substrate. Subsequently, the photoresist was removed for more isotropic etching; the 2nd DRIE isolated and sharpened microneedles from the predefined trench structures. Depending on diverse photomask designs, the 2nd DRIE formed arrays of microneedles that have various height distributions, as well as diverse cross-sectional shapes across the substrate. With these simple steps, high-aspect ratio microneedles were created in the high density of up to 625 microneedles mm^−2^ on a Si wafer. Insertion tests showed a small force as low as ~ 172 µN/microneedle is required for microneedle arrays to penetrate the dura mater of a mouse brain. To demonstrate a feasibility of drug delivery application, we also implemented silk microneedle arrays using molding processes. The fabrication method of the present study is expected to be broadly applicable to create microneedle structures for drug delivery, neuroprosthetic devices, and so on.

## Highlights

- High-density out-of-plane microneedle arrays were fabricated with a single photolithography and two deep reactive ion etching (DRIE) steps in anisotropic and isotropic modes, respectively.
- Microneedles in various heights were monolithically created by the identical DRIE processes and scanning electron microscopy images showed extremely sharp sub-micron (~145-nm-wide) tip.
- Diverse cross-sectional shapes of microneedles were implemented by altering photomask patterns.

## Graphical abstract

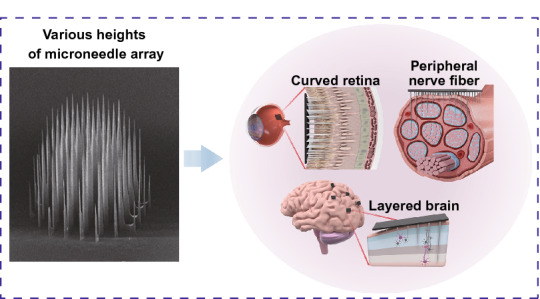

## Introduction

High-aspect ratio microneedle structures have long been studied for various applications. For example, it has been well known that micro-sized needle arrays can deliver drugs without pain that would be caused by the insertion of macro-sized syringe needles [1]. It is because microneedles can reach down to the epidermis layer at a limited penetration depth without irritating dermis layers associated with pain and tissue damage [2, 3]. Thus, microneedles in various shapes have been used for transcutaneous delivery of diverse drugs [4–8] even including recombinant COVID-19 vaccines [9, 10]. Recent publications have also shown that microneedles can be applied to blood vessels [11], vesicles [12], corneas [13], heart [14], and plants [15, 16], demonstrating a possibility of a much broader array of applications. Furthermore, microneedle structures have been widely used in numerous researches of modern neuroscience and neural engineering [17]. Particularly, neural probes in a shape of out-of-plane microneedle arrays are critical for recording various neural signals ranging from single unit activities [18, 19] to electroencephalogram (EEG) signals [20]. In addition to the electrical recording of neural activities, microneedle arrays can be used for the electrical stimulation of nerve cells in the central/peripheral nervous systems [21–23]. These examples suggest that there are and will be increasing demands on microneedle arrays as neural interface devices.

In both applications (*i.e.*, neural interface and drug delivery), the following three aspects of microneedles can be considered. First, high-density microneedle arrays would be preferred for more effective performance. For instance, high-density microneedle electrodes (*i.e.*, high-channel counts) would better discern complex neural signals from densely populated neurons or nerve fibers, enabling more sophisticated functions of brain machine interfaces (BMIs) due to increased selectivity [17, 21, 24]. Also, there have been numerous literature works reporting the high density of microneedles in diverse fabrication methods to enhance the drug delivery efficiency [3, 25–27]. Second, microneedles in various heights (or lengths) would enable more comprehensive interrogation of neurons located at different depths of the complex three-dimensional nervous systems such as laminated cortex areas [28] and peripheral nerves [22, 29]. In case of electric stimulation as well, convoluted shapes of microelectrode arrays are required for specific applications such as nerve fiber or retinal stimulations [30, 31]. This is because the central/peripheral nervous system consists of three-dimensional structures with several layers which have distinctive functions [22, 32, 33]; microneedles in various heights can stimulate those different layers of a nervous system individually or at once. Third, the cross-sectional shape of microneedles would be also of importance for the effective insertion to various target tissues such as brain or skin. Particularly, difficulties in device insertion of high-density microneedles often limit appropriate performances: for example, a pneumatic inserter was needed to implant Utah arrays to the brain tissue [34, 35] because numerous microneedles as a whole require big penetration forces. It has been known that the necessary insertion force can be substantially reduced with a smaller diameter [36] and/or increased sharpness of microstructures [37–39]. Moreover, a sharp tip of neuroprosthetic devices is preferred to minimize insertion damage of neural tissues [40–43], as well as to reduce foreign-body response [44, 45]. In certain applications, specific cross-sectional shapes would be preferred for reducing the puncture resistance, such as polygonal and star-shaped microneedles [46].

Conventional fabrication methods are not appropriate for monolithic fabrication of high-density out-of-plane microneedle arrays in varying heights and diverse cross-sectional shapes (*i.e.*, satisfying the abovementioned three features at once). For example, the Utah arrays for neural applications are manufactured by a series of steps including dicing of silicon (Si) wafers, and wet etching of resulting Si pillars [30, 47]. Since dicing kerf lines (~ 100 μm) determines both dimension and density of microneedle arrays, the high-density array with small feature size has long been challenging to be obtained. In the Utah arrays, 25 electrodes mm^−2^ seems to be the record to date [23]. For drug delivery applications as well, microneedles have been fabricated by complex etching processes [1, 48] or other methods such as the drawing lithography [49–51]. These existing fabrication technologies are too complex or do not allow various cross-sectional shapes of final microneedle structures which may be beneficial in other applications.

In the present study, we report a novel but simple fabrication method for high-density out-of-plane microneedle arrays that can have various heights in arbitrary distribution and diverse cross-sectional shapes depending on photomask pattern designs. The proposed method consists of one single photolithography and two subsequent deep reactive ion etching (DRIE) processes, which is much simpler than conventional methods, for example, such as that of the Utah array. We demonstrate the density of microneedles can be as high as 625 microneedles mm^−2^ and the aspect ratio of microneedles can be as high as ~ 25. Also, the controllability of microneedle cross-sectional shapes is demonstrated.

## Experimental Methods

### Fabrication Step Overview, Materials, and Chemicals

**Fig. 1:**
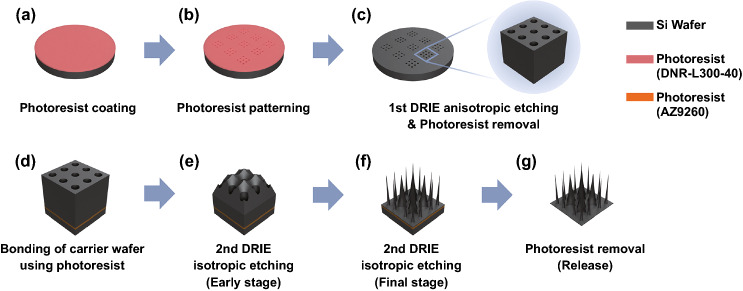
Schematics of fabrication steps of the microneedle arrays. **a** Photoresist (DNR-L300-40) coating on a processing Si wafer. **b** Patterning the photoresist as a masking layer for following deep reactive ion etching (DRIE). **c** Anisotropic etching (the 1st DRIE) of Si and subsequent removal of the photoresist. Inset shows magnified view of microwells formed by the anisotropic etching. **d** Bonding a carrier wafer under the processing wafer using another photoresist (AZ9260). **e**, **f** Isotropic etching (the 2nd DRIE) of Si microstructures. The microstructures at early (*e*) and final (*f*) stages are shown. **g** Release of the fabricated microneedle arrays from the carrier wafer by removing the bonding photoresist

**Fig. 2:**
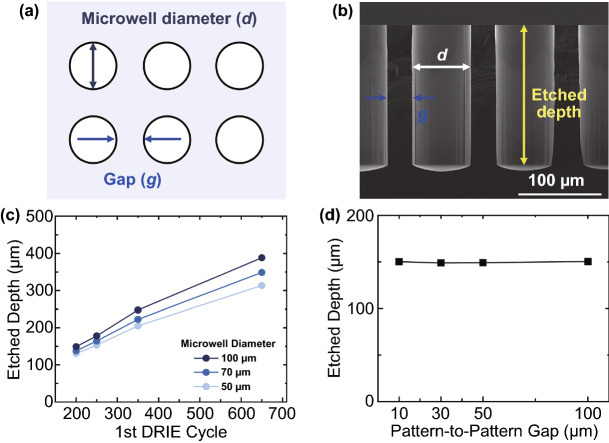
Characterization of the anisotropic etching (the 1st DRIE) process to create microwell structures **a** Microwell diameter (*d*) and pattern-to-pattern gap (*g*) are shown as design parameters of a photomask. **b** A SEM image shows cross-sectional view of the microwells fabricated by 250 cycles of anisotropic etching (*d* = 70 μm and *g* = 30 μm). **c** Etched depths of microwells are plotted as a function of the 1st DRIE cycle numbers for 50, 70, and 100 μm in microwell diameter. **d** Depth of microwells etched by 250 DRIE cycles as a function of pattern-to-pattern gaps ranging from 10 to 100 μm. Microwell diameter was fixed at 50 μm

**Fig. 3:**
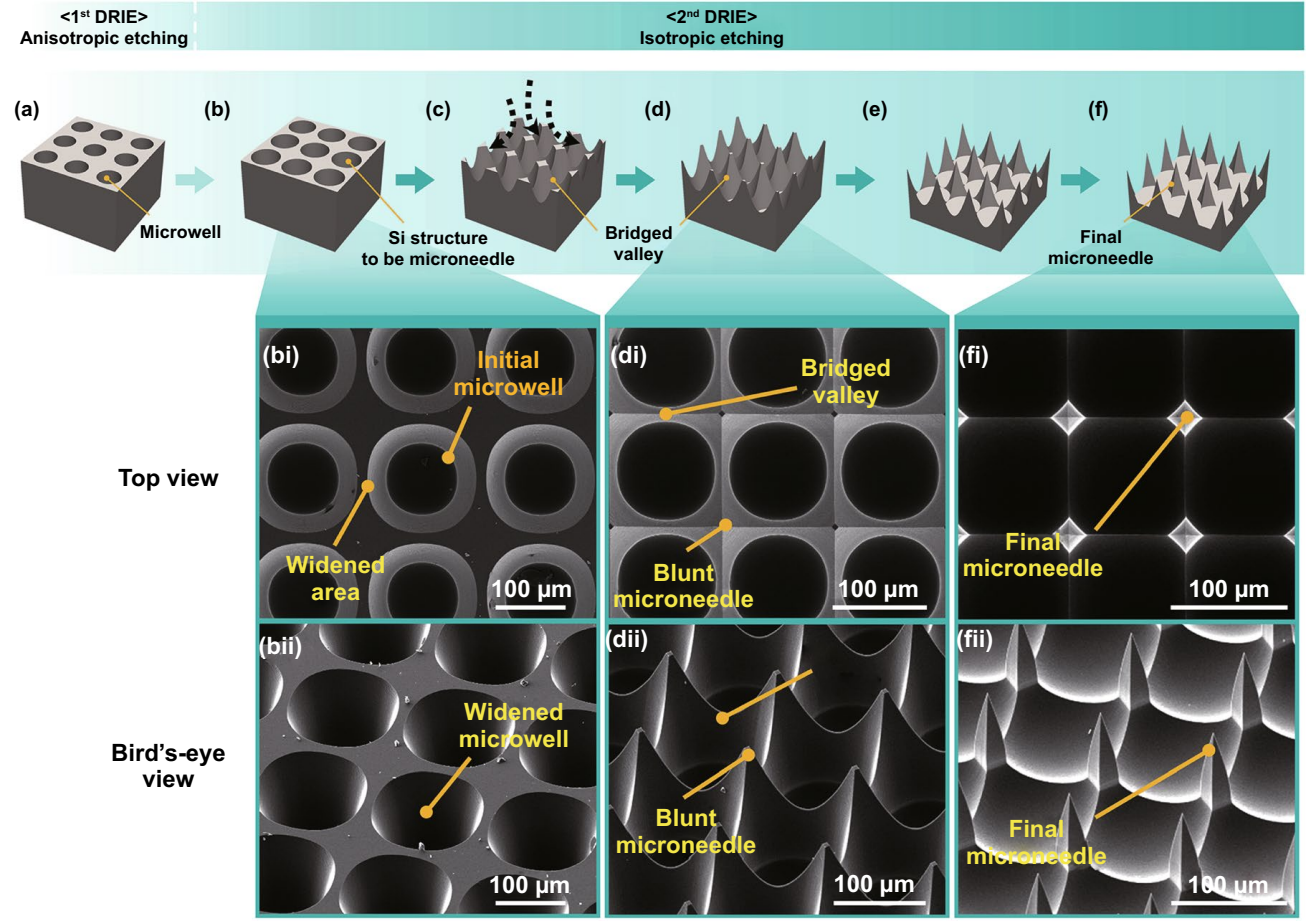
Schematics and SEM images of Si geometries during the isotropic etching (the 2nd DRIE) process to sharpen microneedles. **a** Fabricated microwells after the 1st DRIE step. **b** Early stage of the 2nd DRIE: Initial microwells are widened due to isotropic etching. **c**, **d** Intermediate stage: Adjacent widened microwells connected to each other, forming blunt microneedles and bridged valleys. **e**, **f** Final stage: Sharp microneedles are produced and the bridged valleys are gradually disappeared. SEM images in top view and bird’s-eye view are shown at the bottom of schematics for early (*bi*,* bii*), intermediated (*di*,* dii*), and final (*fi*,* fii*) stages

**Fig. 4:**
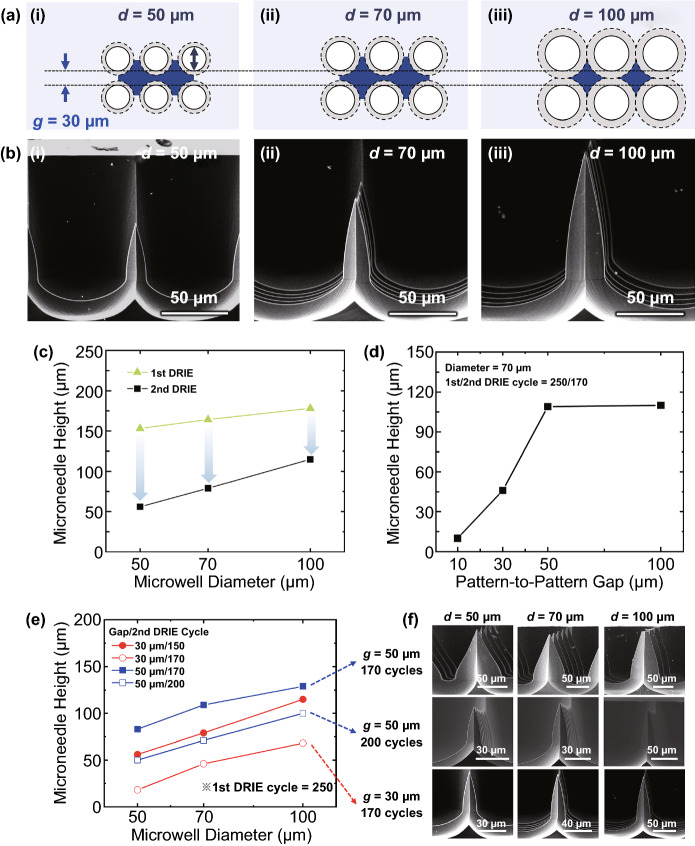
Effects of the design parameters and cycles of isotropic etching on final microneedle structure. **a** Illustration of photomask designs with a fixed gap (*g*) of 30 μm and microwell diameters (*d*) of 50, 70, and 100 μm. Solid and dashed circles indicate initial microwell patterns after the anisotropic DRIE and widened areas during the isotropic DRIE, respectively. Blue polygons represent Si areas to be microneedles. **b** SEM images showing cross-sectional views of microneedles created from the three microwell diameters. **c** Microneedle heights as a function of different microwell diameters after the 1st and the 2nd DRIEs. **d** Microneedle heights as a function of pattern-to-pattern gap. **e** Microneedle heights as function of microwell diameters for several combinations of pattern-to-pattern gap and number of the 2nd DRIE cycles. **f** SEM images of microneedles fabricated from the three conditions described in panel *e*. Note that, scale bars are different in each SEM image

**Fig. 5:**
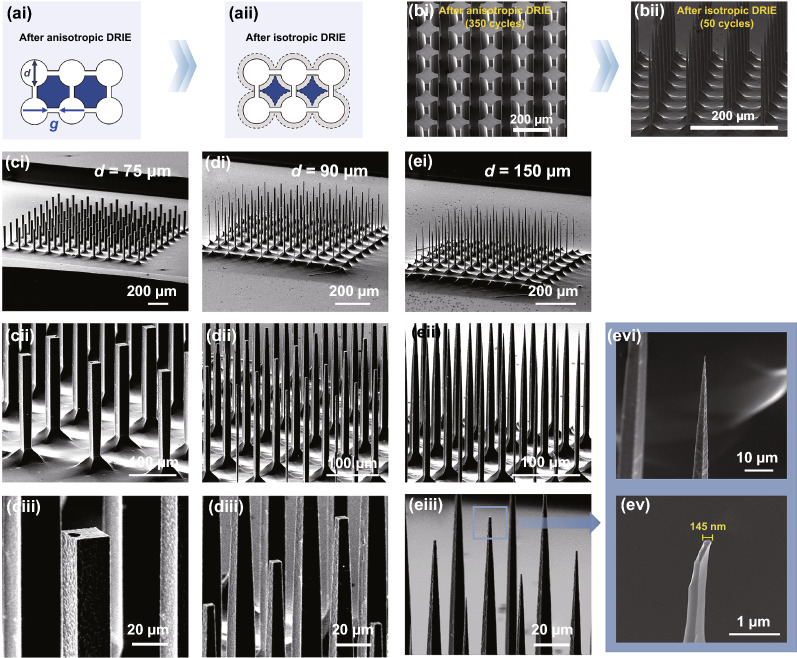
Fabrication of high-aspect ratio microneedles using dumbbell well photomask patterns. **a** Illustration of dumbbell well pattern designs after anisotropic and isotropic DRIE steps. **b** SEM images of anisotropic (350 cycles) and isotropic (50 cycles) process. **c**–**e** SEM images of microneedles created from dumbbell diameters of 75, 90, and 150 μm. Enlarged SEM images show the tip of microneedles from the 150-μm-wide dumbbell well pattern in panel *e* (*evi* and *ev*). The width of microneedle tip was about 145 nm

**Fig. 6:**
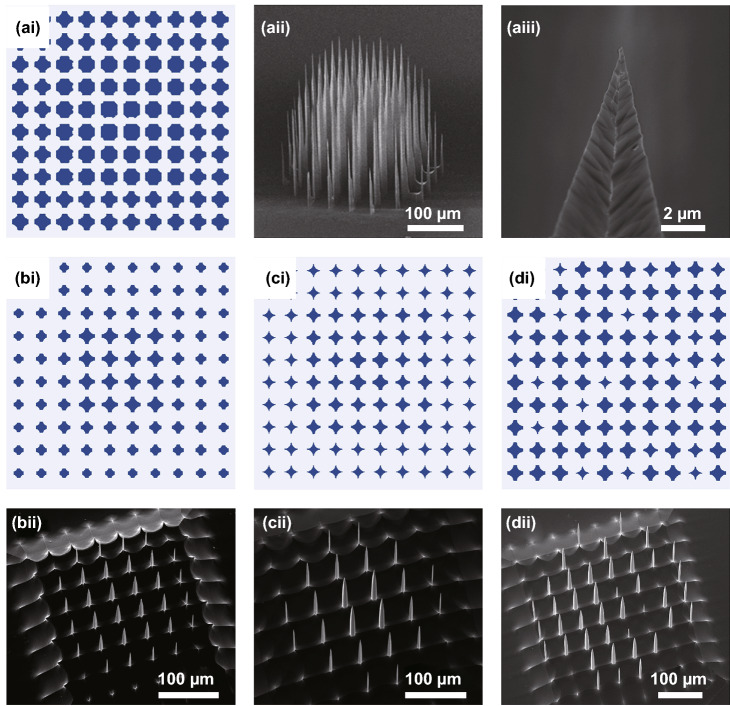
Monolithic fabrication of microneedles in various height distributions on a single wafer. **a** Photomask design for a convex microneedle array (*ai*) and SEM images of the fabricated convex microneedle array (*aii* and *aiii*). **b**–**d** Photomask design for shorter (*bi*) and lower density (*ci*) convex microneedle arrays, and microneedles with an irregular height distribution (*di*). SEM images of the fabricated microneedle arrays (*bii-dii*) from those photomask designs

Figure 1 illustrates the fabrication process flow used in the present study for creating microneedle arrays. A four-inch silicon (Si) wafer (*p*-type <100>, 525 μm in thickness) was used as a processing wafer. Another Si wafer in the same specification was used as a carrier wafer during the 2nd DRIE step. We used the conventional DRIE Bosch process and the detail conditions of each step are summarized in Table 1. For completely isolated microneedles, a silicon-on-insulator (SOI) wafer was used, which had 350, 1, and 100 μm in thicknesses for handling Si, buried oxide, and device Si layers, respectively (later in Fig. 8). To have final microneedle arrays, our fabrication processes had one photolithography which was followed by two DRIE steps: (1) photoresist coating on a Si substrate (Fig. 1a), (2) photolithography for patterning the photoresist (Fig. 1b), (3) anisotropic etching of Si using DRIE and photoresist removal (Fig. 1c), (4) bonding to a carrier wafer using a photoresist layer (Fig. 1d), (5) isotropic etching of Si using DRIE (Fig. 1e, f), and (6) removal of the bonding photoresist for release (Fig. 1g). All fabrication steps of the proposed method were performed without any wet etching. Acetone, isopropyl alcohol, and all other solvents were purchased from Dae-Jung Chemicals (Seoul, Republic of Korea).

**Table 1:** Process parameters of deep reactive ion etching for one single DRIE cycle

	Passivation step	Breakthrough etch step	Main etch step
Time (s)	7	9	14
Step overlap (s)	0.5	1	0
Gas flow ratio of C_4_F_8_:SF_6_:O_2_	120:0:0	25:130:13	0:10:1
Pressure (mTorr)	94	94	15
RF coil power (W)	800	2500	600
RF platen power (W)	0	20	0

### Photolithography and Anisotropic Etching

DNR-L-300–40 (Dongjin Semichem Inc., Seoul, Republic of Korea) was patterned to serve as a masking photoresist layer for the 1st DRIE step. First, the photoresist (DNR-L-300-40) was spin-coated at a thickness of 5.5 μm on a processing wafer (Fig. 1a). After soft baking on a hot plate, the coated photoresist was exposed to UV light using a mask aligner (MA6, SÜSS MicroTec SE, Garching, Germany). After post-exposure bake on the hot plate, the processing wafer was soaked in the fresh developer solution, AZ 300 MIF, and then agitated and rinsed with deionized water (Fig. 1b). After the photolithography, deep microwell arrays were formed on the Si substrate by the conventional DRIE processes (Fig. 1c) using an Omega® LPX-DSi Etch system (SPTS Technologies Ltd., Newport, United Kingdom). Then, the masking photoresist was removed (Fig. 1c) using the O_2_ plasma asher (V15-G, PINK GmbH Thermosysteme, Wertheim, Germany). This anisotropic etching defined shapes and distributions of microwells depending on the photomask designs, which will eventually determine the final geometries of the microneedles. The purpose of the photoresist as a bonding material was to increase isotropic feature during the DRIE process. It is conjectured that the bonding photoresist layer hindered the cooling of the processing wafer [43], as well as diminished the straight movement of biased ion [52–54]. Interestingly, the enhanced isotropic feature during the DRIE process was not observed with the crystal bond which is typically used as bonding materials for effective cooling during DRIE processes.

### Isotropic Etching with Photoresist Bonding

The Si structures remained after the anisotropic DRIE were further isotropically etched to form microneedles. After the anisotropic etching, another photoresist AZ 9260 (Merck KGaA, Darmstadt, Germany) was used to bond a carrier wafer underneath the processing wafer for the following 2nd DRIE: a layer of AZ 9260 was spin-coated in 10 μm on a bare (carrier) wafer. Then, the processing wafer was bonded onto the carrier wafer on a hot plate at 110 °C (Fig. 1d). Si structures more isotropically etched during the 2nd DRIE step (Fig. 1e, f). The final microneedle structures were released from the carrier wafer by removing the bonding photoresist (Fig. 1g). For removing the photoresist layer, AZ 300 MIF (Merck KGaA, Darmstadt, Germany) and acetone were used.

### Characterization of Microneedles

Scanning electron microscopy (SEM; Nova Nano SEM 450, FEI, Eindhoven, Netherlands) was performed to measure dimensions of etched microwell arrays after the 1st DRIE, as well as to characterize the structure of microneedle arrays after the 2nd DRIE. Also, to assess the penetration capability of microneedles, insertion forces to agarose gel tissue phantoms and a mouse brain were measured using a digital force gauge (M5-012E, Mark-10, Copiague, NY, USA) placed on a motorized test stand (ESM303E, Mark-10). The agarose gel phantoms were prepared in two different concentrations (0.5% and 1%) by mixing agarose powder (Sigma-Aldrich, St. Louis, MO, USA) in a phosphate buffered solution. It has been known that ~ 0.5% agarose gel mimics the compression mechanics of the brain [36] but we also doubled concentration (1%) of agarose gel to additionally verify the penetration capability of microneedles to a harder sample.

An adult mouse (C57BL/6 strain, 8 weeks old) was anesthetized by intraperitoneal injection of urethane (1.5 g kg^−1^) and then euthanized by cervical dislocation for the acute experiment. The middle of the mouse head was shaved, and its skull was drilled to expose the brain surface. The animal experiment protocol was approved (KIST-2020-166) by Institutional Animal Care and Use Committees of KIST.

### Transfer Molding of Microneedles

To demonstrate a feasibility of creating microneedles in different materials, we used an array of Si microneedles as the first mold and a polydimethylsiloxane (PDMS; Dow Corning Corporation, Midland, MI, USA) layer as the second mold (as shown later in Fig. 12). First, the surface of microneedles was coated with 50 μL trichloro (1H,1H,2H,2H-perfluorooctyl) silane (PFOTS, Sigma-Aldrich, St. Louis, MO, USA) for 2 h inside a vacuum desiccator to promote later release between the Si microneedles and the PDMS layer. Next, the PDMS, which was mixed with a curing agent at a ratio of 10:1, was poured onto the microneedles and cured for 2 h at 80 °C. Subsequently, a solution of 7% silk fibroin was poured onto the prepared PDMS mold. Finally, the silk microneedle array was peeled off from the PDMS mold.

The silk fibroin solution was prepared as follows: (1) a silkworm cocoon (13.75 g; Uljinsilk, Gyeongsangbuk-do, Republic of Korea), sodium oleate (1.03 g), sodium carbonate (0.69 g) were heated with distilled water (350 mL), (2) after 1 h, the mixture was filtered and washed several times with distilled water, (3) the filtered mixture was heated again with 700 mL of distilled water for 1 h and stored overnight at room temperature, (4) a chemical reactor containing the dried mixture and 120 mL of lithium bromide (LiBr) was placed in a 70 °C water bath for 1 h with an overhead stirrer rotating at 300 rpm, (5) the silk mixture was dialyzed for 2 days, and (6) the dialysis was verified by using a pocket salt meter and the mixture filtered using a Kimtech wipe and stored overnight at room temperature. All chemicals were used as purchased without further alteration.

## Results and Discussion

### Anisotropic Etching Creates Microwell Structures

The control of microneedle heights would be essential to make microneedles adequately reach target depths [2, 55]. In our fabrication approaches, the height control begins with the 1st anisotropic DRIE step. In Fig. 2a, photomask patterns which determined microwell areas to be etched are shown in white circles with black solid lines. After the 1st DRIE process, the cross section of resulting deep microwells was imaged and their depths were measured in the SEM image (Fig. 2b). To further accurately characterize our DRIE process, we plotted etching depths as a function of DRIE cycles (Fig. 2c). For all microwell diameters (*d*) we tested, the depth of etched microwells increased linearly as the number of DRIE cycles increased. Also, consistent with a previous study [56], it appears that a bigger opening in the masking photoresist layer resulted in a deeper trench at a given the number of DRIE cycles. It is thought to be because, due to the higher gas accessibility, the anisotropic DRIE etched Si faster in bigger microwells [57]. For example, in case of the microwell diameter of 100 μm, the etched depth increased from 149 to 388 μm with increasing DRIE cycles from 200 to 650 (darkest blue trace of Fig. 2c). In contrast, in case of the microwell diameter of 50 μm, the etched depth increased from 131 to 313 μm (light blue trace of Fig. 2c). In addition, etch rate difference was more clearly shown for greater numbers of etching cycles: When the diameter of microwell increased from 50 to 100 μm, 200 cycles etched 13% (from 131 to 149 μm) deeper microwells, while 650 cycles etched 23% (from 313 to 388 μm) deeper ones (compare lightest vs. darkest blue symbols in Fig. 2c). Interestingly, the pattern-to-pattern gap (*g*) did not show any substantial change on the etched depth of microwells (Fig. 2d). Our results suggest that low-density microneedle arrays which have larger etching areas would require smaller numbers of DRIE cycles.

### Additional Isotropic Etching Produces Microneedle Structures

To create sharp microneedles at the area surrounded by neighboring four microwells, we performed another DRIE step which was designed to promote isotropic etching of the Si layer by removing the masking photoresist and using another photoresist to reduce substrate cooling (see Sect. 2). Figure 3 shows how the silicon structures gradually changed as the isotropic etching progressed, eventually becoming the sharp microneedles. Due to the enhanced lateral etching, the early stage of isotropic etching widened the microwells (Fig. 3b, bi, and bii). When the adjacent widening microwells touched each other, those interfacial areas were further vertically etched to form bridged valleys (Fig. 3c, d, di, and dii). As the isotropic etching continued, the heights of bridged valleys decreased and the blunt tips of microneedles were sharpened (*compare* Fig. 3dii vs. fii).

Next, we examined the effects of a couple of photomask design parameters on the geometries of final microneedle structures after both DRIE steps (Fig. 4). First, we compared microneedles created from three different microwell diameters (*d*), while the pattern-to-pattern gap (*g*) was fixed at 30 μm (Fig. 4a). Gray areas with dashed lines of Fig. 4a represent widening parts of microwells during the 2nd isotropic DRIE step; this widening eventually created microneedles in the areas highlighted in blue. For the photomask patterns with all three diameters, we performed the same numbers of 1st and 2nd DRIE cycles (250 and 150, respectively), and then, we obtained longer microneedles from bigger microwells (Fig. 4b). This unique feature (*i.e.*, microneedle height controllability by varying microwell diameters) enabled us to monolithically incorporate microneedles in various heights even with identical DRIE processes (see Sect. 3.7 and Fig. 6). Quantitatively, the final heights of microneedles were 56, 79, and 115 μm from microwells of 50, 70, and 100 μm in diameter, respectively (black trace of Fig. 4c). It seems like there were two factors that come into play: (1) earlier anisotropic etching was faster in wider microwells (see Fig. 2d and green curve of Fig. 4c), and (2) the relatively smaller size of micropillars was over-etched than bigger ones during the isotropic DRIE (black curve of Fig. 4c). Regarding the second factor, in case of 100-μm-wide microwells, for example, the 150 cycles of isotropic etching process were proper to fabricate long and sharp microneedles; for 50-μm-wide microwells, however, the same number of DRIE cycles shortened the microneedles by design to have different lengths of microneedles nearby even after enough sharpening (*compare* Fig. 4biii and 4bi). Given the fact that 250 cycles of the 1st DRIE did not make big differences in etching depth (*see* green line in Fig. 4c), the aforementioned second factor seems more critical in making microneedle height differences dependent on the microwell diameter (*see* black line in Fig. 4c).

Different from the anisotropic etching (Fig. 2e), the pattern-to-pattern gap affected the microneedle height during the isotropic etching (Fig. 4d): the microneedle height increased up to 50 μm of the gap, but the height was saturated beyond that point. These results were also caused by the over-etching effect due to the different thicknesses (or widths from the top view) of remaining micropillars after the 1st DRIE step: Smaller gaps created narrower micropillars which were also vertically etched faster. Then, we also varied both pattern-to-pattern gaps and numbers of 2nd DRIE cycles but with the fixed 1st DRIE cycles of 250 (Fig. 4e). First, when we increased the 2nd DRIE cycles from 150 to 170 for microwell patterns separated by 30 μm gaps, microneedles heights were substantially reduced for all microwell sizes (*compare* red lines in Fig. 4e). For instance, the microneedle height created from 100-μm-wide microwells decreased significantly from 115 to 68 μm because of the over-etching effect. Similarly, the pattern-to-pattern gap of 50 μm also decreased the microneedle height with increased isotropic etching cycles (*compare* blue lines in Fig. 4e). However, this reduction in height is unavoidable to obtain the sharp microneedle. The final microneedle structures obtained from different conditions were summarized in a series of SEM images (Fig. 4f): If the pattern-to-pattern gap was increased from 30 to 50 μm with 170 cycles of isotropic etching, the sharpness of microneedles decreased along with increasing their microwell diameter size. For example, in the first row of Fig. 4f, the microneedle from 100 μm microwell shows a dull shape compared to other 50 and 70 μm microneedles. It seems like these longer but dull microneedles were resulted from insufficient isotropic etching. Accordingly, when the 2nd DRIE cycle of the 50 μm-gap microwells was increased to 200, the microneedles became sharper (SEM images shown in the second row of Fig. 4f). It is interesting to note that both overall shape and sidewall profiles of those microneedles were remarkably similar to the microneedles which were fabricated from 30 μm-gap microwells and isotropically etched during 170 cycles (*compare* SEM images shown in the second and third rows of Fig. 4f). Taken together, our results suggest that the isotropic etching process initially contributed a way of sharpening the microneedles, but it rather decreased the height of microneedles after a certain number of cycles depending on pattern-to-pattern gaps. For more optimization of fabrication processes to precisely adjust the target height and shape of the microneedle, a comprehensive understanding of the effects of the microwell diameter, the 1st/2nd DRIE cycles, and the pattern-to-pattern gap would be needed in addition to our results described above.

### High-Aspect Ratio Microneedles Using Dumbbell Well Photomask Patterns

Although the aspect ratio of the fabricated microneedles can be modulated by varying design and process parameters (Fig. 4), the achievable aspect ratio of the microneedles using the circular microwell array was pretty low (~ 3.2). It was thought to be because of the bridged valley formed between neighboring microneedles (Fig. 3dii), which should be minimized during the 2nd DRIE process; however, the continuous isotropic etching resulted in shorter microneedles, reducing their aspect ratio. Increasing diameters of microwells can enhance the aspect ratio but make pitches bigger, lowering the density of the microneedle array. To achieve both high-aspect ratio and high density of microneedles, we devised a dumbbell-shaped microwell photomask pattern (Fig. 5a). We connected adjacent microwell patterns to each other to make microneedle-to-be areas isolated even from the first anisotropic etching (blue area; Fig. 5a). After the 1st DRIE (350 cycles), which created the micropillars (Fig. 5bi), the subsequent 2nd DRIE (50 cycles) turned the individual micropillars into slender microneedles with sharp tips (Fig. 5bii). The SEM images in Fig. 5c–e show the representative microneedles fabricated after 350 and 50 cycles of the 1st and 2nd DRIEs for the different diameters of microwells. Due to the different microwell sizes, the microneedle base width was decreased from 16 to 8 µm, while the microwell diameter changed from 75 and 150 μm. Also, the same isotropic etching did not introduce any further sharpness for the microwells of 75 μm in diameter (Fig. 5ciii), while dull and extremely sharp microneedles were fabricated from the microwells of 90 and 150 μm, respectively (Fig. 5diii and eiii). Because the dumbbell well patterns did not produce any bridge valleys much smaller, number of 2nd DRIE cycles was enough to isolate microneedles, minimizing over-etching. As a result, the highest aspect ratio (~ 25) was obtained from the dumbbell well diameter of 150 µm. Also, those microneedles were remarkably slender, demonstrating the ratio between base and top widths of ~ 1.9. The magnified SEM image also reveals extremely sharp sub-micron (~ 145-nm-wide) tip at the very end of microneedles (Fig. 5evi and ev).

### Microneedles with Irregular Height Distribution

In neuro-engineering applications, an array of microneedle electrodes in various length can enable researchers access neural information processed in both vertical and horizontal directions [58]. However, it has been challenging to monolithically incorporate out-of-plane microneedles (e.g., Utah probe) of different heights (or lengths) although convoluted [30] or slanted [23] electrode arrays had been demonstrated. To cover variable penetration depths in 3D neural tissues such as cortex, retina, and nerve fiber, it would be necessary to monolithically integrate microneedles in various heights in a precisely designed manner.

In Sect. 3.2, we demonstrated the microneedle length modulation as a function of the microwell diameter designed in photomask (Fig. 4). With this unique feature of our proposed method, diverse heights of microneedles were obtained in a single Si wafer using identical fabrication processes but different photomask designs, which have various diameters and distributions of dumbbell wells (Fig. 6). First, to have convex shape of the surface defined by microelectrode tips, we placed 30 µm of small dumbbell wells at the center of the photomask and made the diameters of wells gradually increased up to 40 µm with decreasing gap from 20 to 10 µm as the photomask pattern moved outward (Fig. 6ai). Indeed, the fabricated microneedle array had a convex tip contour with longer microneedles located at the center and shorter microneedles at the perimeter of the sample (Fig. 6aii). In this photomask design, the microneedle heights ranged from 68.54 to 246.44 µm, resulting in the biggest difference of 177.90 µm. Theoretically, the height deviation across microneedles in a given array can be further increased by using thicker processing wafers. A magnified view of SEM image clearly shows the apex of the microneedle has a sub-micron tip (Fig. 6aiii).

To show other examples for diverse microneedle profiles, we designed photomasks that have different dumbbell well diameters and pattern-to-pattern gaps (Fig. 6bi, ci, and di) and fabricated microneedle arrays using those photomasks (Fig. 6bii, cii, and dii). For instance, in case of Fig. 6bi and ci, we increased the diameters and decreased gap of dumbbell wells than those of Fig. 6ai, creating shorter and low-density convex shape microneedles (*compare* Fig. 6bii and cii vs. Fig. 6aii). When the pattern-to-pattern gap was changed irregularly (Fig. 6di), microneedles with irregular heights were produced (Fig. 6dii). These examples suggest a possibility that our proposed method can be further expanded to fabricate microneedle arrays which have other height distributions in a designed manner.

### High-Density and Completely Isolated Microneedle Arrays

**Fig. 7:**
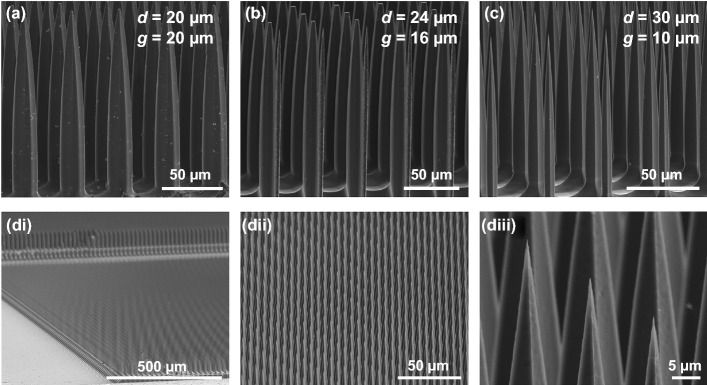
High-density microneedle arrays using the dumbbell well patterns. **a**–**c** SEM images of final microneedle structures fabricated from different design parameters. Dumbbell well diameter (*d*) and pattern-to-pattern gap (*g*) are shown at the top right. **d** SEM images of the high-density microneedle array (*di-dii*) and enlarged view showing sharp tip (*diii*). The design parameters were same as panel *c* (*d* = 30 μm, *g* = 10 μm)

Another important design feature of the microneedle array is density. As a penetrating neural interface, higher density microneedle would access densely populated neurons or nerve fibers individually, resulting in a more sophisticated investigation of the mechanism underlying complex spatial neural interactions. To explore whether this high density is achievable, we designed dumbbell well arrays that have three different combinations of microwell diameters and gaps (Fig. 7a–c). Figure 7d shows microneedle arrays fabricated in the highest density from the same photomask pattern with Fig. 7c: It has 15,625 microneedles in 5 × 5 mm^2^ of a Si wafer (Fig. 7di), corresponding to the microneedle density of 625 microneedles mm^−2^. The final microneedle structures were also extremely uniform across the array (Fig. 7dii) and had remarkably sharp tips (Fig. 7diii). In contrast to wet etching processes which showed considerable variations in microneedle lengths [23], our proposed method using DRIEs demonstrated <  ~ 1.2% variations from target heights. Also, the width of microneedles at 90% of the total length from the bottom varies ~ 1.4% at most, further supporting the good uniformity of our fabrication method. We further confirmed that these high-density microneedle arrays could be successfully fabricated with all three kinds of different dumbbell well diameters (Fig. 7). Given the length of the microneedles, the high-density sharp microneedles shown in Fig. 7 might be a good candidate for intra-retinal stimulation [59] to differentially activate ON and OFF pathways of the retina [60, 61].

**Fig. 8:**
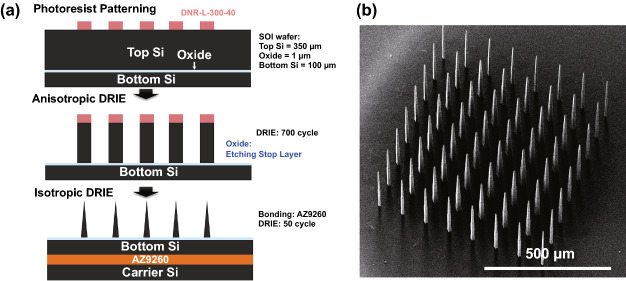
**a** Fabrication process flows using a silicon-on-insulator (SOI) wafer for complete isolation of microneedles. The oxide layer of SOI wafer acts as an etching stop layer during anisotropic etching to eliminate bridge valleys which appear with bulk Si wafers (see Fig. 3). **b** SEM image of a final microneedle array fabricated with a SOI wafer

We observed that bulk Si wafers left small hillocks at the base of microneedles (Fig. 5). A silicon-on-insulator (SOI) wafer can be used to create a flat microneedle base. Similar to the fabrication processes for bulk Si wafers, we first patterned a photoresist layer (first row of Fig. 8a) but we used the SOI wafer upside down to etch the handle layer to obtain microneedles as long as possible. The main advantage of the use of SOI wafers appeared during the 1st DRIE step (second row of Fig. 8a): We were able to over-etch the top Si layer (700 cycles for 350 μm for the SOI wafer we used) since the oxide layer acted as a good etch stop layer without damaging the bottom Si layer. Finally, individual microneedles which had a precise height same as the thickness of Top Si layer were created. Then, after the masking photoresist removal, a carrier wafer was bonded under the SOI wafer. Lastly, 50 cycles of the DRIE process were performed for isotropic etching (third row of Fig. 8a). The SEM image of the final microneedles showed neither bridge valleys nor hillocks at the microneedle bases (*compare* Fig. 5cii and 8b).

### Insertion Force Analysis

**Fig. 9:**
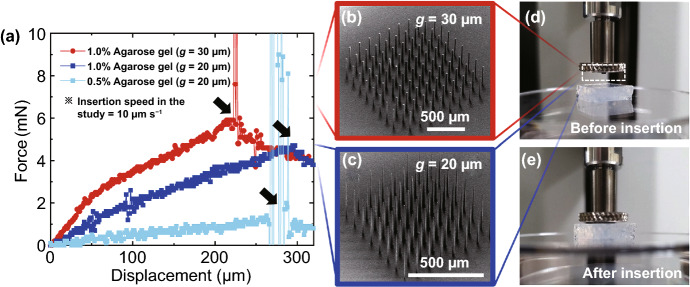
Insertion tests of the fabricated microneedle arrays to agarose gels mimicking brain tissues. **a** Force–displacement response during the insertion of microneedle arrays into 0.5% and 1% agarose gels at an insertion rate of 10 μm s^−1^. **b, c** SEM images of the microneedle arrays used in the insertion tests, which were fabricated from 30 and 20 μm gap sizes (for *b* and *c*, respectively). **d, e** Photographs of the experiments taken before (*d*) and after (*e*) insertion of a microneedle array

To evaluate the feasibility of practical use of the fabricated sharp microneedles, we measured the required force to insert microneedles into tissue phantoms and a mouse brain. As a reference, the smallest insertion force of microneedles to human skin reported so far is 10 mN [48], which is still 500 times bigger than the force used by a mosquito to insert its labium (18 μN) [62]. With the microneedles fabricated from SOI wafers which had approximately ~ 25 and ~ 10 μm in diameters (created from a gap of 30 and 20 µm, respectively; Fig. 9b, c), the insertion forces were characterized quantitatively (*see* Sect. 2). Figure 9a shows the measured forces as a function of displacement when microneedle arrays were being inserted to 1.0% or 0.5% agarose gels at a speed of 10 µm s^−1^ (Fig. 9d, e). During pre-penetration insertion stage, the microneedles contacted the surface of agarose gels and the monotonic increments of the measured forces were observed until the penetration occurred as highlighted with arrows in Fig. 9a. After the peak point of each plot which shows the different puncture force and displacement, the force was suddenly dropped, indicating post-penetration relaxation stage [62]. As aforementioned, the sharpness of the microneedle is correlated with the design parameter such as the gap of the microwell array (Fig. 4d); the tips of the microneedles with the 20 µm gaps were ~ 1.72 μm which were ~ 6 times sharper than those with the 30 µm gaps (Fig. 9b, c). It is worth noting that the insertion force was smaller for the sharper microneedles (*compare* red and blue traces in Fig. 9a): When inserting a microneedle array fabricated with the 30 µm gaps into the 1.0% agarose gel, the penetration force was about 6 mN. On the other hand, when inserting a microneedle array fabricated with the 20 µm gaps into the same agarose gel, it required a ~ 21% lower force. These results clearly indicate that the sharper tip of microneedle arrays requires less insertion force, which is consistent with previous work [36]. When inserting the sharper microneedle array into this softer tissue phantom (0.5% agarose gel), the array could be inserted with less insertion force which was about 1.4 mN (light blue trace in Fig. 9a).

**Fig. 10:**
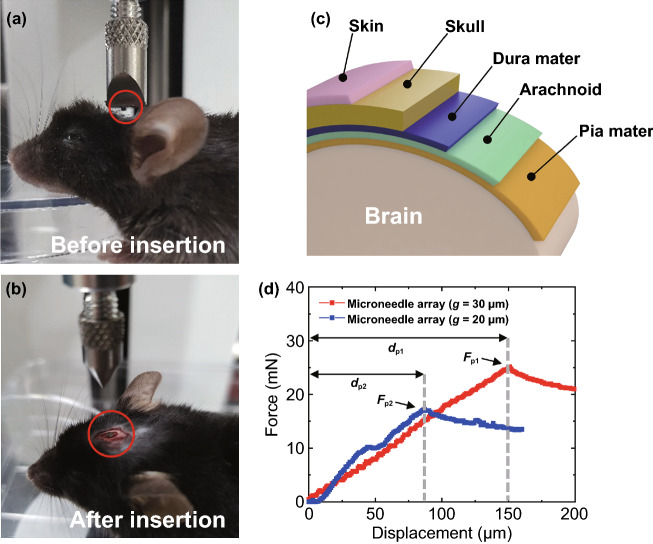
Insertion tests of the fabricated microneedle arrays to a mouse brain. **a, b** Microneedle array was positioned above the bregma and completely penetrated the brain. A microneedle array is shown in a red circle in both panels. **c** Schematic diagram showing layers of a mouse brain. In this experiment, skin and skull were removed but other layers were remained. **d** Force–displacement plot during the insertion of microneedle arrays with a speed of 10 μm s^−1^ into a mouse brain. We used the two microneedle arrays shown in Fig. 9b, c: the red and the blue traces show measured forces as a function of loading displacement for dull and sharp microneedles, g = 30 and 20 μm, respectively. Puncture force (*F*_p_) and displacement (*d*_p_) were characterized for each array of microneedles. The vertical dashed gray lines divide pre- and post-penetration periods. (Color figure online)

As previously reported, since the agarose gel cannot completely replace real biological tissue [36], we also tested the fabricated 10 × 10 microneedles array using a mouse brain (Fig. 10a, b). The mouse brain was exposed by removing skin and drilling the skull at a bregma position, but the dura mater was not removed (Fig. 10c). The experiment was conducted while the mouse head was fixed under the digital force gauge (*see* Experimental Methods). In Fig. 10d, we plotted measured forces as a function of loading displacement for both dull and sharp microneedle arrays shown in Fig. 9b, c. During the dull microneedles compressed the surface of the dura mater with up to ~ 150 μm displacement before puncture (*d*_p1_), the force was linearly increased (pre-penetration dimpling stage caused by insertion force; red trace of Fig. 10d). At the moment of penetrating the dura mater, the puncture force (*F*_p1_) was measured to be ~ 25.2 mN, which is corresponding to 252 µN per one microneedle. This result indicates that even the slightly dull 100 microneedles needed ~ 10 mN smaller insertion force than that of one single Si microprobe inserted to a rat brain (~ 35 mN) [63], demonstrating a good penetration capability of our microneedle arrays. As expected from our previous experiment using the agarose gels, the array of sharper microneedles (which is created with pattern gap of 20 μm) needed about ~ 32% smaller insertion force to penetrate the dura mater than the dull microneedles. In detail, the sharper microneedles showed a lower insertion force: The puncture force (*F*_p2_) was measured to be ~ 17.2 mN, which is corresponding to 172 µN per each microneedle (blue trace of Fig. 10d). This insertion force of the sharp individual microneedle is comparable to that of a caterpillar spine to penetrate the mouse skin (~ 173 µN) [62]. Also, compared to the neural probe inserted to a rat brain [63], each sharp microneedle we fabricated required just about one two-hundredth (*i.e.*, ~ 0.5%) force. In both arrays of microneedles, after the puncture of the dura mater, the force was rapidly decreased (post-penetration relaxation stage; Fig. 10d), similar to the measurements performed in the agarose gels (Fig. 9a). Interestingly, the insertion force of the sharp microneedles was more sophisticated, showing less monotonic increment including small bump at ~ 50 µm of displacement. The discrepancy between the two force profiles is highly likely due to the different sharpness of the microneedle arrays. For instance, as suggested longer displacement before puncture (*d*_p1_), the dull sample might have a deeper dimple before the penetration and then break dura mater and arachnoid at the same time to reach pia mater. On the other hand, the sharp sample might have two steps of penetration: (1) first puncture of dura mater, and (2) another puncture of arachnoid until they reach down to the pia mater. We did not investigate these possibilities further due to the limited resolution of our force gauge.

### Diverse Cross-Sectional Shapes of Microneedles

The existing fabrication technologies typically produce microneedles in circular cross sections such as conical or cylindrical shapes [5, 23] and usually do not allow various cross-sectional shapes of final microneedle structures. Since the shape of microneedles is directly related to the stress applied to the skin or brain tissue, the development of various types of microneedles would broaden our understanding of penetrating mechanics as a scientific tool [2, 46, 48]. For example, a previous study reported, with optimized cross-sectional shapes, can minimize the contact area with the skin, resulting in more reduced puncture resistance and easier insertion [46]. In addition, microneedles in various cross-sectional shapes may be attractive in other applications such as drug delivery [48] and superhydrophobic surfaces [64]. As another scientific tool, microneedle arrays can be used to monitor the physical properties of cell culture platforms [65]. Further applications may include field emission display using Fowler–Nordheim tunneling effect if nanometer scale apex can be realized.

**Fig. 11:**
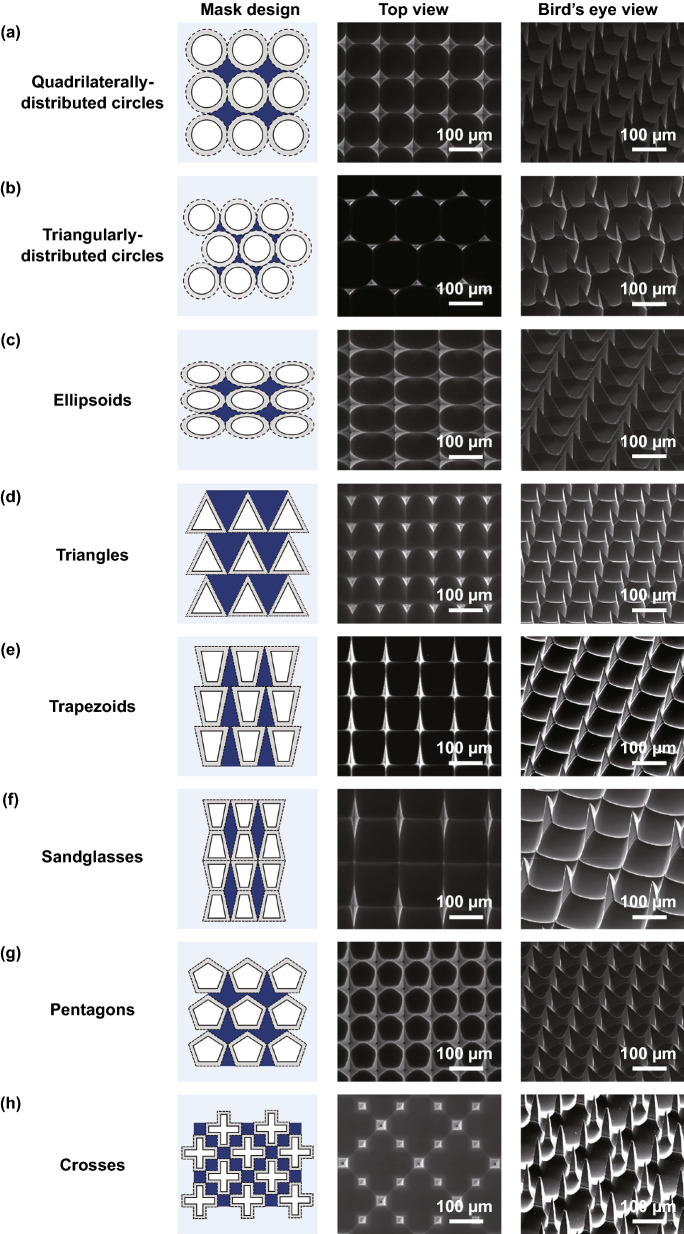
Fabrication of microneedles that have diverse cross-sectional shapes from various photomask designs. Mask patterns, top and bird’s-eye view SEM images are shown for **a** quadrilaterally distributed circles, **b** triangularly distributed circles, **c** ellipsoids, **d** triangles, **e** trapezoids, **f** sandglasses, **g** pentagons, and **h** crosses

Interestingly, in our proposed approaches, the tip sharpness and the sidewall profile of microneedles were controllable by the photomask design which define areas to be anisotropically etched (Fig. 4e). Thus, we further explored whether cross-sectional shapes of microneedles could be altered by photomask patterns. To implement various cross-sectional shapes of microneedle arrays, we tested eight different photomask designs which have distinct microwell opening patterns and their spatial distributions (Fig. 11; quadrilaterally distributed circles, triangularly distributed circles, ellipsoids, triangles, trapezoids, sandglasses, pentagons, and crosses, respectively). Among them, the pattern of quadrilaterally distributed circles was the one used for fabricating microneedles described in previous sections. In each photomask design summarized in Fig. 11, microneedles were formed in the areas highlighted in blue. SEM images in top views and bird’s-eye views of Fig. 11 show various microneedle shapes; the final cross-sectional shape of microneedles can be easily controlled by the photomask designs such as pattern shapes and distribution of those shapes. For instance, even with the same size of circles, the final structures of microneedles were substantially distinct depending on their quadrilateral/triangular distribution (Fig. 11a, b). These examples suggest that more intriguing shapes of microneedles can be fabricated by altering photomask designs.

### Fabrication of Silk Microneedles by Transfer Molding

**Fig. 12:**
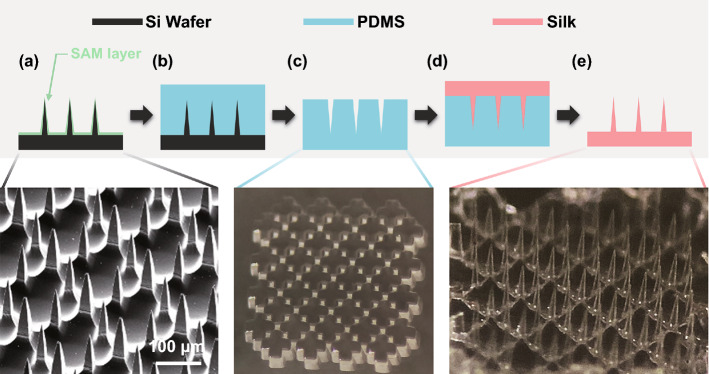
Fabrication of silk microneedles by transfer molding techniques. **a** Self-assembled monolayer (SAM) coating on a Si microneedle array. A SEM image of microneedles fabricated from the photomask pattern of crosses is shown at the bottom. **b** Pouring polydimethylsiloxane (PDMS) elastomer on the Si microneedles. **c** Detachment of PDMS layer from the Si microneedles. A photograph of the created PDMS mold is shown at the bottom. **d** Pouring prepared silk solution onto a PDMS replica. **e** Detachment of the silk microneedles from the PDMS mold. A photograph of the final silk microneedles is shown at the bottom

To demonstrate a possibility to be used as a drug delivery platform or biodegradable microneedle patches [10, 66], we explored whether our Si microneedle arrays could be expanded to other polymeric materials using a molding process [67]. To promote the releasing process during soft-lithography, a self-assembled monolayer was formed on microneedles created from the crosses pattern (Fig. 12a). The 10:1 polydimethylsiloxane elastomer (PDMS; Dow Corning Corporation, Midland, MI, USA) was poured on the prepared Si microneedle template (Fig. 12b). After curing, the PDMS replica of the microneedles slowly detached from the Si wafer (Fig. 12c). Next, using the same method with PDMS replica, silk microneedles were fabricated by pouring prepared silk solution (*see* Methods) on the PDMS mold (Fig. 12d). As shown in Fig. 12e, the initial structure of Si microneedles was successfully transferred to silk microneedles. The transfer molding process can be applicable with other types of biodegradable [68, 69] and biocompatible materials [70]. Also, further height modulation of microneedles is expected to enable more effective drug delivery to the epidermis or upper dermis layer by penetrating the stratum corneum without pain [2, 48, 71].

## Conclusions

In the present study, we have demonstrated the fabrication of Si microneedle arrays that have various cross-sectional shapes and irregular height distributions by using single photolithography and two subsequent DRIE steps. The first highly anisotropic DRIE step after photolithography created microwells as an array in a Si wafer. In the 2nd DRIE step, more isotropic etching isolated and sharpened microneedles subsequently. The proposed method which used dry etching processes enabled us to achieve the high density (625 microneedles mm^−2^) of microneedles in a single array, as well as the highest aspect ratio (~ 25). Both density and aspect ratio may be even more enhanced by optimizing fabrication processes. Also, the final cross-sectional shapes of microneedles were controlled by the shape and distribution of areas to be etching in the 1st DRIE and could be further altered by simply modifying the photomask design. Moreover, the convex/irregular profile of tips of microneedles that have various heights in a given array was fabricated. Those arrays of microneedles in variable lengths can reach various depths of the skin or the brain to promote more effective with adequate penetration depths. It is expected to be more useful if those microneedles are monolithically packaged with integrated circuits fabricated on a solid Si substrate to access highly curved tissues. By insertion test of microneedles using agarose gels and a mouse brain, the required insertion force was characterized. The fabricated microneedle array required only 172 µN per one microneedle to penetrate the mouse brain. Taken all together, our new fabrication method can facilitate the manufacture of microneedle arrays for various applications including drug delivery, neurophysiological/neuroprosthetic researches, and so on. The use of mature semiconductor fabrication processes is expected to enable the creations of microneedles in a large area.
